# Integrated miRNA profiling and bioinformatics analyses reveal potential causative miRNAs in gastric adenocarcinoma

**DOI:** 10.18632/oncotarget.5419

**Published:** 2015-10-07

**Authors:** Xiaojing Zhang, Yin Peng, Zhe Jin, Weiling Huang, Yulan Cheng, Yudan Liu, Xianling Feng, Mengting Yang, Yong Huang, Zhenfu Zhao, Liang Wang, Yanjie Wei, Xinmin Fan, Duo Zheng, Stephen J. Meltzer

**Affiliations:** ^1^ Department of Pathology, The Shenzhen University School of Medicine, Shenzhen, Guangdong, People's Republic of China; ^2^ Department of Medicine/GI Division, Johns Hopkins University and Sidney Kimmel Cancer Center, Baltimore, MD, USA; ^3^ Shenzhen Key Laboratory of Micromolecule Innovatal Drugs, The Shenzhen University School of Medicine, Shenzhen, Guangdong, People's Republic of China; ^4^ Shenzhen Key Laboratory of Translational Medicine of Tumor, The Shenzhen University School of Medicine, Shenzhen, Guangdong, People's Republic of China; ^5^ Laboratory of Chemical Genomics, School of Chemical Biology and Biotechnology, Peking University Shenzhen Graduate School, Shenzhen, Guangdong, People's Republic of China; ^6^ School of Pharmacy, China Medical University, Shenyang, Liaoning, People's Republic of China; ^7^ Department of Pathology, Wuhan University School of Basic Medical Sciences, Hubei, People's Republic of China; ^8^ Center for High Performance Computing, Shenzhen Institutes of Advanced Technology, Shenzhen, Guangdong, People's Republic of China

**Keywords:** gastric cancer, miRNA profiling, bioinformatics

## Abstract

Gastric cancer (GC) is one of the leading causes of cancer-related deaths throughout China and worldwide. The discovery of microRNAs (miRNAs) has provided a new opportunity for developing diagnostic biomarkers and effective therapeutic targets in GC. By performing microarray analyses of benign and malignant gastric epithelial cell lines (HFE145, NCI-N87, MKN28, RF1, KATO III and RF48), 16 significantly dysregulated miRNAs were found. 11 of these were validated by real-time qRT-PCR. Based on miRWalk online database scans, 703 potential mRNA targets of the 16 miRNAs were identified. Bioinformatic analyses suggested that these dysregulated miRNAs and their predicted targets were principally involved in tumor pathogenesis, MAPK signaling, and apoptosis. Finally, miRNA-gene network analyses identified miRNA-125b as a crucial miRNA in GC development. Taken together, these results develop a comprehensive expression and functional profile of differentially expressed miRNAs related to gastric oncogenesis. This profile may serve as a potential tool for biomarker and therapeutic target identification in GC patients.

## INTRODUCTION

Gastric cancer (GC), one of the most common malignancies worldwide, is also the primary cause of cancer-related deaths in China and internationally [1]. Due in part to the absence of early diagnostic modalities, GC is usually diagnosed at late stages, with a very poor prognosis. The 5-year survival rate of late-stage GC is only 3.1%, whereas the 5-year survival of early GC is up to 90% [2]. Therefore, it is critical to further elucidate the molecular pathogenesis of GC in order to identify molecular biomarkers for early detection and novel targets for effective therapy.

MicroRNAs (miRNAs) are small, single-stranded RNAs that negatively modulate transcription by sequence-specific interaction with the 3′ untranslated regions (UTRs) of target mRNAs [3]. Their target genes are involved in many cancer-related cellular processes, including cell cycle regulation, differentiation, apoptosis, and invasion/migration. MiRNAs have been characterized as oncogenes or tumor suppressors [4]. Dysregulated expression of miRNAs is a newly proposed mechanism of cancer pathogenesis [5, 6]; specific miRNAs are associated with each type of tumor development and progression [7, 8]. Nevertheless, the unique expression profiles of miRNAs and their downstream signaling pathways in GC remain incompletely characterized. Therefore, the purpose of the current study was to delineate the global miRNA expression profile and related signaling networks in GC.

MiRNA microarray analyses were first performed to identify dysregulated miRNAs in human GC cell lines. 16 miRNAs were found to be aberrantly expressed, 11 of which were chosen and validated by real-time qRT-PCR miRNAs-106a, -17, -20a, -92a, -100, -125b, -127-3p, -145, -381, and -455. Results of these qRT-PCRs agreed with miRNA microarray data. 2,532 potential mRNA targets of these miRNAs, related to cell proliferation, cell death, apoptosis, and known vital tumorigenesis signaling pathways, were predicted by further bioinformatics analyses based on the online miRWalk database. In addition, miRNA-gene network analyses suggested miRNA-125b as a key miRNA in GC pathogenesis.

## RESULTS

### Identification of dysregulated miRNAs

**Figure 1:**
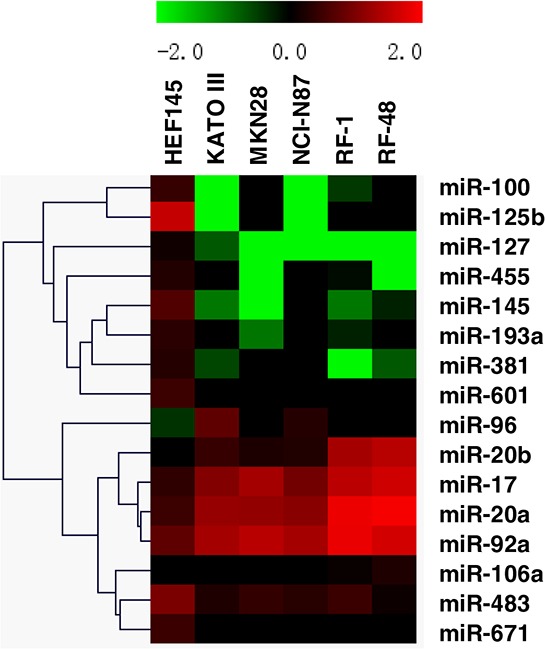
Alterations in miRNA expression profiles across 5 gastric cancer cell lines and 1 normal gastric cell line

**Figure 2:**
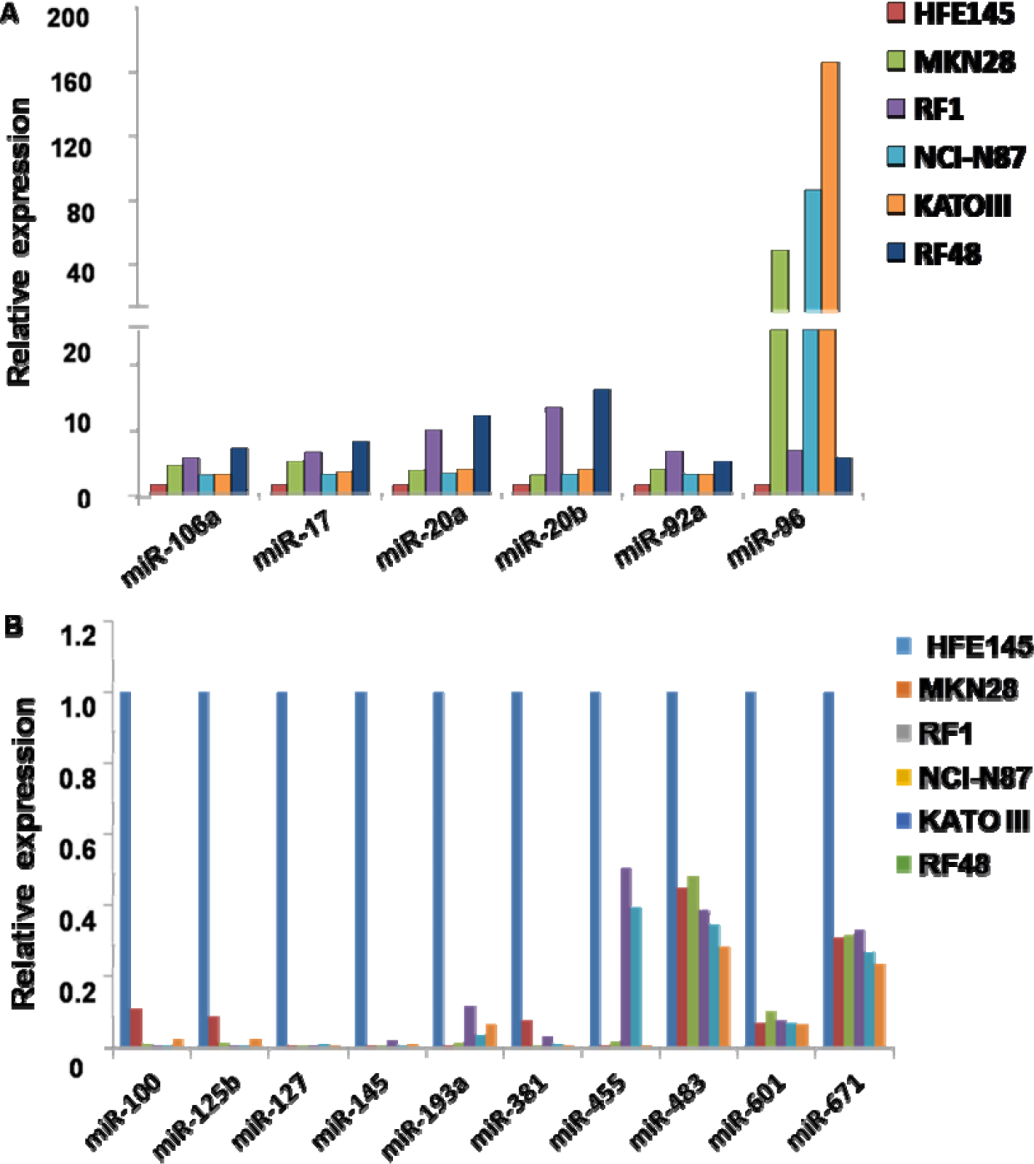
MiRNAs upregulated and downregulated in all 5 gastric cancer cell lines by microRNA microarray **A.** Expression patterns of upregulated miRNAs; **B.** Expression patterns of down-regulated miRNAs. MiRNAs in Figures A and B were identified based on *a* ≥ 2-fold change in signal intensity on microarrays.

To better understand miRNA dysregulation in GC cells, comprehensive miRNA expression profiles were assessed in two groups of gastric cell lines by microRNA microarray. The expression patterns of normal gastric cells (HFE145) and GC cells (NCI-N87, MKN28, RF1, KATO III and RF48) were distinct (Figure 1). MiRNAs exhibiting two-fold or greater differences between all 5 GC cell lines and normal cells are shown in Supplementary Tables S1 and S2. In total, 16 significantly dysregulated miRNAs were identified from microarray analyses. Relative to the expression profile of the normal gastric cell line HFE145, 6 miRNAs (miRNAs-106a, -17, -20a, -20b, -92a and -96) were consistently overexpressed, while 10 miRNAs (miRNAs-100, -125b, -127, -145, -193a, -381, -455, -483, -601, and -671) were uniformly downregulated in all 5 GC cell lines (Figure 2).

### Validation of miRNA expression by qRT-PCR

**Figure 3:**
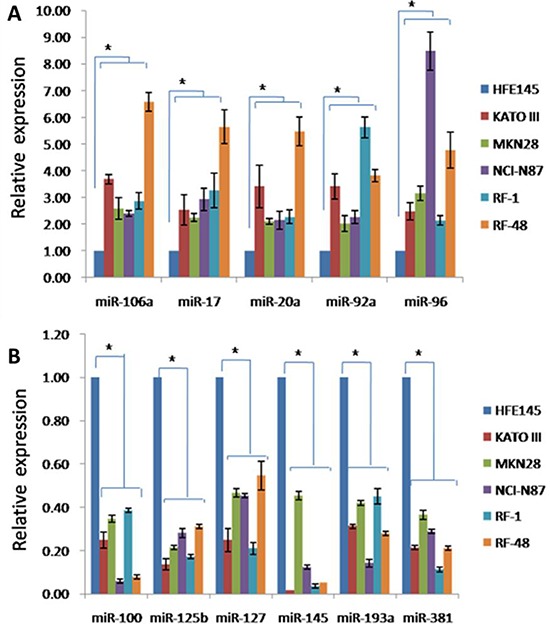
Validation of miRNA expression alterations in GC cells **A.** Expression levels of upregulated miRNAs in human GC cells determined by qRT-PCR. **B.** Expression levels of downregulated miRNAs in human GC cells determined by qRT-PCR. *P* < 0.05.

To validate miRNA microarray data, qRT-PCRs were performed using TaqMan MicroRNA Assays. 11 miRNAs (miRNAs-106a, -17, -20a, -92a, -96, -100, -125b, -127-3p, -145, -193a, -381) were randomly selected for validation from the 16 significantly dysregulated miRNAs. Expression levels of all 11 miRNAs by qRT-PCR agreed consistently with miRNA microarray results. Among these 11 miRNAs, 5(miRNAs-106a, -17, -20a, -92a and -92a) and 6(miRNAs -100, -125b, -145, -127, -193a and -381) were upregulated or downregulated more than 2-fold in all 5 GC cell lines, respectively (Figure 3). These results showed that gastric tumorigenesis was characterized by significant changes in miRNA expression profiles.

### GO analysis

To elucidate functions of dysregulated miRNAs in GC, potential mRNA targets of altered miRNAs were collected using the Validated Target module of miRWalk. 2,532 targets were obtained. Among these targets, deduplication was conducted and 703 (27.7%) targets were finally saved.

**Figure 4:**
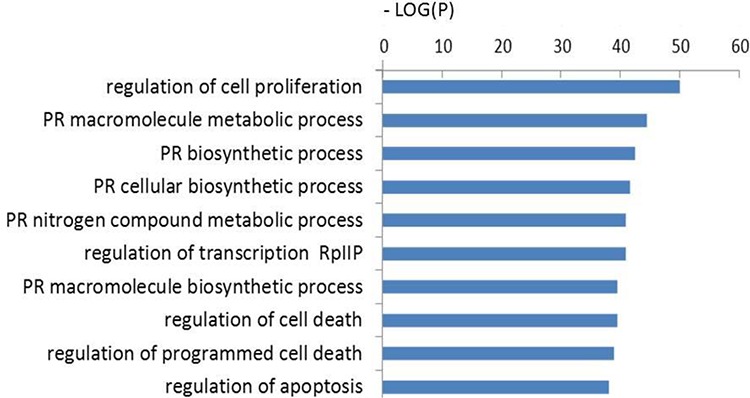
GO analysis of candidate target genes Functional annotations of 16 differentially expressed miRNAs. −Log (*P*) values for negative logarithm of *P* value, greater value represents more enrichment of significance. PR: positive regulation; RpIIP: RNA polymerase II promoter.

The functions of these 703 target genes were then analyzed using GO annotation with the DAVID tool. When setting the cutoff standard at *P* < 0.01 and FDR < 0.05, 446, 27 and 33 GO terms, respectively, were found from three ontologies: biological processes, cellular components and molecular functions (Table 1). The 10 most significant GO terms are shown in Table 1. In addition, cluster analysis was also conducted to search for the enrichment of targets represented by the negative logarithm of the *P* value (−Log (*P*)) in the ontology of biological processes. The top 10 functional classes are shown in Figure 4. According to cluster analyses for biological functions, most of the predicted targets of altered miRNAs were involved in cell proliferation, transcription, cell death, programmed cell death, RNA metabolic process, and gene expression. These functions are known to be strongly associated with human tumorigenesis and metastasis [9].

**Table 1:** The enriched GO categories of predicted targets genes (The top 10, FDR < 0.05, *P* < 0.01)

Term	Count	*P* Value	Fold Enrichment	FDR
**Most significant GO biological processes(total GO term = 446)**				
GO:0042127∼regulation of cell proliferation	149	1.77E-50	3.934269826	3.29E-47
GO:0010604∼positive regulation of macromolecule metabolic process	148	5.77E-45	3.588669796	1.07E-41
GO:0009891∼positive regulation of biosynthetic process	130	5.33E-43	3.886969687	9.87E-40
GO:0031328∼positive regulation of cellular biosynthetic process	128	3.01E-42	3.88304125	5.58E-39
GO:0051173∼positive regulation of nitrogen compound metabolic process	123	1.59E-41	3.968915476	2.95E-38
GO:0006357∼regulation of transcription from RNA polymerase II promoter	131	1.66E-41	3.744462545	3.08E-38
GO:0010557∼positive regulation of macromolecule biosynthetic process	122	4.48E-40	3.876454478	8.31E-37
GO:0010941∼regulation of cell death	137	5.13E-40	3.493136562	9.50E-37
GO:0043067∼regulation of programmed cell death	136	1.60E-39	3.48045069	2.96E-36
GO:0042981∼regulation of apoptosis	134	1.16E-38	3.463389657	2.15E-35
**Most significant GO cellular component (total GO term = 27)**				
GO:0031981∼nuclear lumen	142	9.86E-23	2.313779081	1.41E-19
GO:0031974∼membrane-enclosed lumen	161	1.48E-20	2.049507218	2.11E-17
GO:0043233∼organelle lumen	158	3.70E-20	2.051101948	5.29E-17
GO:0005654∼nucleoplasm	100	4.74E-20	2.678754804	6.79E-17
GO:0070013∼intracellular organelle lumen	149	1.85E-17	1.978845413	2.65E-14
GO:0005667∼transcription factor complex	38	9.81E-14	4.275292668	1.40E-10
GO:0005829∼cytosol	107	4.97E-11	1.900788014	7.11E-08
GO:0044451∼nucleoplasm part	59	1.28E-10	2.511658424	1.83E-07
GO:0045121∼membrane raft	26	1.27E-09	4.295748614	1.82E-06
GO:0009986∼cell surface	42	2.43E-09	2.851488304	3.48E-06
**Most significant GO molecular function (total GO term = 33)**				
GO:0030528∼transcription regulator activity	178	1.16E-31	2.441568622	1.79E-28
GO:0003700∼transcription factor activity	130	1.63E-27	2.765282215	2.53E-24
GO:0043565∼sequence-specific DNA binding	98	8.85E-27	3.348405977	1.37E-23
GO:0016563∼transcription activator activity	76	1.90E-24	3.844416738	2.95E-21
GO:0046983∼protein dimerization activity	82	1.61E-20	3.137727975	2.49E-17
GO:0003702∼RNA polymerase II transcription factor activity	49	2.67E-17	4.164923008	4.15E-14
GO:0046982∼protein heterodimerization activity	43	1.26E-15	4.287516896	1.90E-12
GO:0003677∼DNA binding	192	2.58E-15	1.708282449	3.96E-12
GO:0008134∼transcription factor binding	69	1.59E-14	2.789539077	2.46E-11
GO:0019899∼enzyme binding	68	1.33E-13	2.696546711	2.06E-10

### KEGG pathway analysis

**Figure 5:**
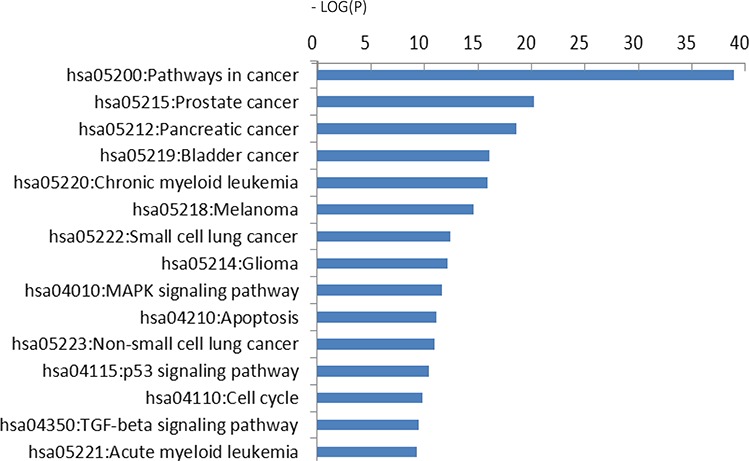
KEGG pathway analysis Predicted targets of 16 significantly differentially expressed miRNAs. The vertical axis is the pathway category; the horizontal axis is the enrichment of pathways represented by −Log (*P*) values.

To gain a better understanding of the functions and regulatory networks of predicted target genes, we searched for target enrichment in KEGG. There were 702 differentially expressed genes identified in 29 pathways with a cutoff standard of *P* < 0.01 and FDR < 0.05 by KEGG pathway analysis (Table 2). These results revealed that the predicted targets of dysregulated miRNAs were associated with multiple tumor types, including bladder, pancreatic, lung and colorectal cancer. Predicted targets were correlated with apoptosis, cell cycle, and cell adhesion. Some important signaling pathways known to mediate tumorigenesis and metastasis were also involved, including the TGF-β, p53 and ErbB signaling pathways. Moreover, the MAPK cell cycle pathways were enriched according to–Log (*P*) values (Figure 5).

**Table 2:** The enriched pathways of the predicted target genes (FDR < 0.05, *P* < 0.01)

Pathway ID and Name	genes	*P* Value	FDR
hsa05200:Pathways in cancer	98	1.41E-38	1.69E-35
hsa04010:MAPK signaling pathway	53	4.51E-12	5.41E-09
hsa05215:Prostate cancer	38	2.01E-20	2.42E-17
hsa04510:Focal adhesion	38	3.57E-08	4.29E-05
hsa05212:Pancreatic cancer	33	7.48E-19	8.98E-16
hsa04110:Cell cycle	32	2.70E-10	3.24E-07
hsa05220:Chronic myeloid leukemia	31	3.40E-16	4.00E-13
hsa05218:Melanoma	29	5.96E-15	7.19E-12
hsa05222:Small cell lung cancer	29	8.35E-13	1.00E-09
hsa04722:Neurotrophin signaling pathway	29	1.96E-08	2.35E-05
hsa04210:Apoptosis	28	1.48E-11	1.77E-08
hsa04620:Toll-like receptor signaling pathway	27	3.37E-09	4.05E-06
hsa04660:T cell receptor signaling pathway	27	1.56E-08	1.87E-05
hsa04350:TGF-beta signaling pathway	26	5.49E-10	6.59E-07
hsa05214:Glioma	25	1.36E-12	1.64E-09
hsa05210:Colorectal cancer	25	1.40E-09	1.68E-06
hsa05219:Bladder cancer	24	2.06E-16	2.66E-13
hsa04115:p53 signaling pathway	24	7.07E-11	8.48E-08
hsa04012:ErbB signaling pathway	24	1.62E-08	1.94E-05
hsa05223:Non-small cell lung cancer	22	2.27E-11	2.72E-08
hsa05221:Acute myeloid leukemia	21	8.66E-10	1.04E-06
hsa05211:Renal cell carcinoma	19	9.60E-07	0.001151
hsa04520:Adherens junction	19	4.27E-06	0.005122
hsa05213:Endometrial cancer	18	4.12E-08	4.95E-05
hsa04621:NOD-like receptor signaling pathway	17	3.70E-06	0.00444
hsa04920:Adipocytokine signaling pathway	17	1.10E-05	0.013157
hsa04150:mTOR signaling pathway	15	8.87E-06	0.010635
hsa04930:Type II diabetes mellitus	14	1.35E-05	0.016212
hsa05216:Thyroid cancer	13	2.21E-07	2.66E-04

### Potential causative miRNAs in GC

**Figure 6:**
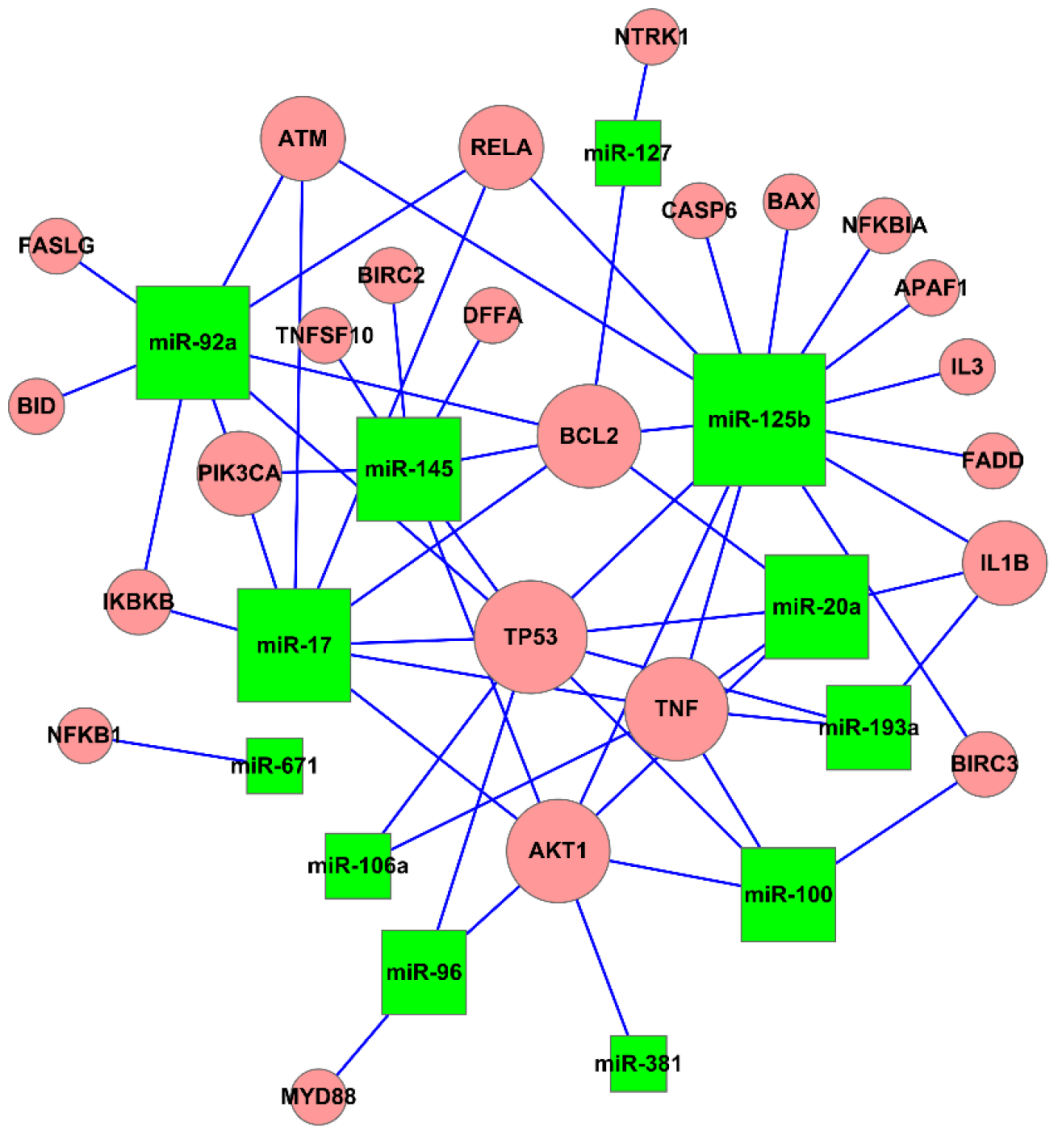
Presumed connections between miRNAs and the target genes The network of apoptosis regulated by the target genes. The size of each node is proportional to the supposed functional connectivity of each miRNA according to numbers of putative target genes. MiRNA-125b is the largest node in the network.

To further elucidate correlations between miRNAs and potential target genes, miRNA-gene network analyses were generated by Cytoscape (Figure 6). Relationships between miRNAs and target genes were analyzed by integrating the top GO analysis results with the KEGG pathway data. In this Figure, the size of each node is proportional to the purported functional connectivity of each miRNA. According to its size in this representation, miRNA-125b is the hub node with the most interactions, suggesting that it is a key causative point within this network.

## DISCUSSION

During the past decade, miRNAs have emerged as key regulators in numerous oncogenic processes. Their discovery has initiated revolutionary progress in cancer research [10–12]. Thus, it is relevant to delineate the expression profiles, functions, and potential regulatory mechanisms of miRNAs in cancers. The current study investigated the expression signature of miRNAs in GC and analyzed their downstream targets, functions, and signaling pathways in GC.

Firstly, miRNA expression profiles were described in gastric cell lines (one NC- and 5 GC-derived cell lines). Compared with normal cells, 6 (miRNAs-106a, -17, -20a, -20b, -92a and -96) and 10 miRNAs (miRNAs-100, -125b, -127, -145, -193a, -381, -455, -483, -601, and -671) were uniformly and significantly overexpressed or downregulated in all 5 GC cell lines, respectively. The differential expression of 11 of these 16 dysregulated miRNAs was validated using RT-qPCR. These results suggest that gastric tumorigenesis is characterized by significant changes in miRNA expression profiles.

Although our microarray data was not validated in primary GC tissue samples, our findings are consistent with previous literature in GC as well as other cancer types. For example, miRNA-106a exhibits oncogenic functions in pancreatic cancer [13]. It was also found to be overexpressed in GC and to regulate invasion and metastasis [14–16]. MiRNAs-17, -20a and -92a, members of the miRNA-17-92 cluster, acting as oncogenes, are also overexpressed in GC tissues [17]. The expression level of miRNA-20b was also found to be increased in GC tissues [18]. Meanwhile, miRNAs-125b, -127, and -145 were downregulated in GC tissues and cell lines. MiRNA-145 was shown to suppress invasion, metastasis, and angiogenesis in GC cells [19–21]. The remaining dysregulated miRNAs that we identified are reported for the first time in GC. Thus, our results provide a rationale for further study of these novel dysregulated miRNAs in gastric tumorigenesis.

Since most miRNAs are believed to be involved in carcinogenesis by inhibiting target mRNAs, we predicted targets of these 16 altered miRNAs. In total, 2,532 targets were identified using the Validated Target module of miRWalk database. Further GO analyses revealed that the majority of predicted target genes functioned in modulating transcriptional regulation, cell proliferation, cell death, and programmed cell death. These functions are known to be closely related to human tumorigenesis and metastasis.

Signaling pathways regulated by validated targets of the 16 dysregulated miRNAs were assessed via KEGG pathway analysis. In the current study, the most significant pathways were closely associated with tumor pathogenesis, including pathways in cancer, pancreatic cancer, prostate cancer, chronic myeloid leukemia, and bladder cancer. In addition to pathways in cancer, the MAPK signaling pathway was also shown to be important in GC development. MAPKs (mitogen-activated protein kinases) principally comprise extracellular signaling–related kinases (ERKs), c-Jun NH2-terminal kinases (JNKs), and p38 MAPKs. These pathways play an important role in cell proliferation and differentiation, survival, apoptosis, and anti-apoptosis [22, 23] and are implicated in various tumor types. Furthermore, Liang *et al*. [24] showed that MAPKs, particularly the ERK subclass, are overexpressed and correlate with tumorigenesis and metastatic potential in GC. In our results, 53 potential mRNA targets were implicated in the MAPK pathway. Thus, elucidating oncogenic mechanisms by which miRNAs regulate the MAPK pathway represents a promising strategy for identifying new therapeutic targets in GC.

Finally, by combining GO terms and KEGG pathway analysis with network analyses, we identified a potential crucial miRNA in gastric carcinogenesis, miRNA-125b. MiRNA-125b can act as either a tumor suppressor or an oncogene, depending on tumor type, with either increased or decreased expression. For example, miRNA-125b expression is decreased in hepatocellular carcinoma (HCC) tissues and HCC cell lines *vs*. normal tissues and cells, suggesting a tumor-suppressive role in this organ [25]. Conversely, in pancreatic cancer, prostate cancer and acute myeloid leukemia, miRNA-125b levels are elevated, implying that it functions as an oncogene in these tissues [26–28]. MiRNA-125b expression levels are heterogeneous in GC cell lines and tissues, according to discoveries by several research groups. Matteo et al [19] found that miRNA-125b levels were significantly downregulated in GC tissues. However, another group showed that miRNA-125b was upregulated in GC (HGC-27, MKN-28, BGC-823, SGC-7901, AGS, and MKN-45 GC cell lines) *vs*. the normal gastric cell line GES-1 [29]. In our study, miRNA-125b was downregulated in all GC cell lines studied (NCI-N87, MKN28, RF1, KATO III and RF48) *vs*. HFE145. Inconsistent findings in previous studies may have been due to histologic heterogeneity in GC. Moreover, previous studies have indicated that expression of miRNA-125b is altered according to pathophysiological conditions [30–32]. For example, in papillary thyroid cancer, miRNA-125b is upregulated, while in anaplastic thyroid carcinoma, expression of miRNA-125b is reduced relative to normal thyroid tissue [32]. Notwithstanding the heterogeneous and controversial expression of miRNA-125b in various cancers, miRNA-125b plays a key role in regulating signaling pathways that mediate cell proliferation, differentiation, and apoptosis [33]. Thus, miRNA-125b may represent a potential biomarker for the diagnosis and independent prognostication of GC. Admittedly, no biological functional experiments have been conducted to elucidate the causative role of miRNA-125b. Nevertheless, further investigations are indicated to overcome this gap in knowledge.

In summary, 16 significantly dysregulated miRNAs were discovered in GC, including 6 up- and 10 down-regulated miRNAs. In addition, 2,532 potential target transcripts were predicted for these 16 miRNAs. Integrated GO and KEGG pathway analyses, combined with miRNA-gene network analyses, uncovered a potential key gastric oncogenic miRNA, miRNA-125b. These findings imply that dysregulated expression of miRNAs and their potential target mRNAs are prominently involved in the pathogenesis of GC, suggesting that dysregulated miRNAs may serve as potential biomarkers and therapeutic targets in GC patients.

## MATERIALS AND METHODS

### Cell lines

Human immortalized normal gastric epithelial cells (NC) HFE145 and GC cells (NCI-N87, MKN28, RF1, KATO III and RF48) were obtained from Howard University and the American Type Culture Collection, respectively. These cells were grown in DMEM medium containing 10% fetal bovine serum (FBS). Cell lines were cultured in a humidified air supplemented with 5% CO_2_ at 37°C.

### Preparation of Total RNA

According to the manufacturer's instructions, total RNA, including the miRNAs, was extracted from cells by using the miRNeasy mini kit (Qiagen, Hilden, Germany). The RNA concentration was measured using a NanoDrop 2000 spectrophotometer (Thermo Fisher Scientific, Waltham, USA). The integrity and the quality of RNA were determined by Agilent 2100 Bioanalyzer (Agilent Technologies, Santa Clara, USA). RNAs with a 2100RIN (RNA integrity number) 6.0 and 28S/18S 0.7 were used for the miRNA array analysis and reverse transcription (RT).

### MicroRNA microarrays

MicroRNA microarray analyses were performed on two groups of cell lines: normal gastric epithelial cells (NC) HFE145 and GC cells (NCI-N87, MKN28, RF1, KATO III and RF48). MicroRNA labeling and microarrays were performed with MicroRNA Labeling Reagent and Hybridization Kits (Agilent, Santa Clara, CA, USA) and Human microRNA Microarray Kits (V2, Agilent), respectively. All procedures were performed as described previously [34]. Every miRNA was represented by 2 probes and 15 replicates on each microarray, which contained 723 human miRNAs and 76 human viral miRNAs. 100 ng of total RNA from each cell line was treated with phosphatase and then labeled with cyanine 3-pCp. Hybridization was carried out according to manufacturers’ instructions. Hybridization signals were detected using an Agilent Microarray Scanner (Agilent) with Agilent's Scan Control version A.7.0.1 software. Quantile normalization was conducted. The value of two probes were averaged and used as the final expression level. Differential analysis was carried out using the Student *t*-test; *P* < 0.05 was set as the cut-off. MiRNAs that were up-regulated or down-regulated by at least two-fold between the two cell line groups were regarded as significantly dysregulated miRNAs.

### Real-time quantitative reverse transcription PCR

Procedures for real-time quantitative reverse-transcription PCR (qRT-PCR) analyses have been described previously [35]. TaqMan MicroRNA Assays, Human (Applied Biosystems, Foster City, CA, USA) and U6 small nuclear RNA TaqMan RT–PCR amplicon (RNU6B TaqMan microRNA Assay kit, Applied Biosystems) were used to confirm miRNA and U6 expression, respectively. U6 was used as an internal control. Fold-change of expression was calculated by using Ct values in comparison with controls. Expression levels were determined by the comparative ΔCt method.

### Target prediction and bioinformatic analyses

In silico prediction of miRNA target genes was carried out by miRWalk (http://www.umm.uni-heidelberg.de/apps/zmf/mirwalk/) [36]. The miRWalk database focuses on predicted as well as validated miRNA binding sites in all known genes in human, mouse and rat. In current study, the potential target genes were processed by the Validated Target module of the miRWalk database. For functional analyses of miRNA potential targets, GO term analysis was applied to organize genes into categories on the basis of biological processes, cellular components and molecular functions [35]. Biological pathways defined by Kyoto Encyclopedia of Genes and Genomes (KEGG) analysis were identified by DAVID (Database for Annotation, Visualization and Integrated Discovery) software (http://david.abcc.ncifcrf.gov/tools.jsp). DAVID online provides a set of functional annotations of a large number of genes. *P*-values of each pathway were adjusted by the method of Benjamini-Hochberg to control the false discovery rate (FDR). In current study, GO terms and signaling pathways were selected with the threshold of significance being defined as *P* < 0.01 and FDR < 0.05.

## SUPPLEMENTARY MATERIAL TABLES


