# Supplementary material for: Integrated miRNA profiling and bioinformatics analyses reveal potential causative miRNAs in gastric adenocarcinoma
Source: Oncotarget. 2015 Oct 7;6(32):32878–89. doi: 10.18632/oncotarget.5419 (PMC4741736; doi:10.18632/oncotarget.5419)
Supplement: Supplementary file 1 [file oncotarget-06-32878-s001.pdf]

SUPPLEMENTARY TABLES

Supplementary Table S1: The up-regulated miRNAs in all 5 gastric cancer cell lines

micro RNA ID	HFE145	KATO III		MKN28		NCI-N87		RF1		RF48	
	Signal	Signal	Fold	Signal	Fold	Signal	Fold	Signal	Fold	Signal	Fold
miR-106a	25.037	85.942	2.301	91.960	3.393	65.760	2.135	112.612	4.255	192.322	5.499
miR-141	1.769	3954.970	1500.000	282.749	147.778	1461.730	672.407	6.070	3.250	9.244	3.741
miR-17	164.864	631.680	2.570	684.702	3.838	448.688	2.214	866.420	4.973	1447.990	6.291
miR-20a	190.216	820.032	2.892	563.358	2.737	560.767	2.398	1538.910	7.656	2503.870	9.423
miR-20b	62.175	269.534	2.906	143.781	2.136	171.546	2.243	684.487	10.416	1101.550	12.685
miR-7	5.172	119.506	15.494	59.438	10.617	52.188	8.206	17.616	3.222	18.082	2.503
miR-92a	284.478	942.559	2.222	867.534	2.818	800.757	2.289	1514.390	5.037	1520.390	3.827
miR-96	1.614	424.449	176.369	89.670	51.339	180.147	90.771	8.750	5.132	9.527	4.229

Supplementary Table S2: The down-regulated miRNAs in all 5 gastric cancer cell lines

microRNA ID	HFE145	KATO III		MKN28		NCI-N87		RF1		RF48	
	Signal	Signal	Fold	Signal	Fold	Signal	Fold	Signal	Fold	Signal	Fold
miR-100	179.752	0.123	0.001	21.251	0.109	−1.893	0.001	1.585	0.008	6.015	0.024
miR-125b	926.690	−0.566	0.000	85.743	0.085	−0.369	0.000	14.002	0.014	31.689	0.024
miR-127	116.849	1.524	0.009	−1.232	0.001	−0.178	0.001	−0.223	0.001	−0.269	0.001
miR-145	243.628	1.065	0.003	−0.406	0.001	6.420	0.021	0.751	0.003	2.715	0.008
miR-193a	155.117	8.176	0.035	0.803	0.005	22.416	0.118	2.069	0.013	14.078	0.065
miR-199a	281.155	−0.332	0.001	−1.067	0.001	−0.454	0.001	−1.406	0.001	−0.646	0.001
miR-210	557.422	83.783	0.101	90.963	0.151	65.854	0.096	44.444	0.075	197.249	0.253
miR-381	143.231	1.913	0.009	11.686	0.075	5.238	0.030	0.183	0.001	1.437	0.007
miR-455	140.382	82.820	0.396	−1.708	0.001	87.143	0.505	2.675	0.018	−0.673	0.001
miR-483	387.521	200.108	0.346	188.557	0.449	183.914	0.386	197.460	0.482	153.485	0.284
miR-601	188.370	19.131	0.068	13.577	0.067	17.506	0.076	20.467	0.103	16.600	0.063
miR-671	187.040	74.876	0.268	62.321	0.308	76.333	0.332	62.693	0.317	61.095	0.234
